# 5,6-Dichloro-1-β-D-ribofuranosylbenzimidazole (DRB) induces apoptosis in breast cancer cells through inhibiting of Mcl-1 expression

**DOI:** 10.1038/s41598-023-39340-x

**Published:** 2023-08-03

**Authors:** Yi-Hsuan Kuo, Tsai-Chun Lai, Chia-Hsin Chang, Han-Ching Hsieh, Feng-Ming Yang, Meng-Chun Hu

**Affiliations:** 1 Graduate Institute of Physiology, National Taiwan University College of Medicine, Taipei, 100 Taiwan; 2 Department of Life Sciences, College of Life Sciences, National Chung Hsing University, Taichung, 402 Taiwan; 3 School of Respiratory Therapy, College of Medicine, Taipei Medical University, Taipei, 110 Taiwan

**Keywords:** Cancer, Drug discovery, Molecular biology

## Abstract

The effective treatment of breast cancer remains a profound clinical challenge, especially due to drug resistance and metastasis which unfortunately arise in many patients. The transcription inhibitor 5,6-dichloro-1-beta-D-ribofuranosyl-benzimidazole (DRB), as a selective inhibitor of cyclin-dependent kinase 9, was shown to be effective in inducing apoptosis in various hematopoietic malignancies. However, the anticancer efficacy of DRB against breast cancer is still unclear. Herein, we demonstrated that administration of DRB to the breast cancer cell line led to the inhibition of cellular proliferation and induction of the typical signs of apoptotic cells, including the increases in Annexin V-positive cells, DNA fragmentation, and activation of caspase-7, caspase-9, and poly (ADP ribose) polymerase (PARP). Treatment of DRB resulted in a rapid decline in the myeloid cell leukemia 1 (Mcl-1) protein, whereas levels of other antiapoptotic proteins did not change. Overexpression of Mcl-1 decreased the DRB-induced PARP cleavage, whereas knockdown of Mcl-1 enhanced the effects of DRB on PARP activation, indicating that loss of Mcl-1 accounts for the DRB-mediated apoptosis in MCF-7 cells, but not in T-47D. Furthermore, we found that co-treatment of MCF-7 cells with an inhibitor of AKT (LY294002) or an inhibitor of the proteasome (MG-132) significantly augmented the DRB-induced apoptosis. These data suggested that DRB in combination with LY294002 or MG-132 may have a greater therapeutic potency against breast cancer cells.

## Introduction

Cyclin-dependent kinases (CDKs) are specific serine (Ser)/threonine (Thr) protein kinases known for playing crucial roles in modulating cell-cycle regulation^1^. Many genes involved in the cell-cycle are frequently mutated in human cancers leading to uncontrolled cell division and growth. Furthermore, several key components of the CDKs machinery are dysregulated in different malignancies^2^. Hence, in the past two decades there has been several studies of CDKs as possible targets for cancer therapy, which led to the development of numerous CDKs inhibitors^3–5^. Despite pharmacologically inhibiting CDKs having been tested in the clinic, the primarily effect was particularly attributed to so for CDK9^6^.

CDK9 is crucial for the proper regulation and progression of transcription through its association with a regulatory cyclin T subunit to form the positive transcription elongation factor b (P-TEFb)^7^. After initiation of transcription, RNA polymerase II is paused by 5,6-dichloro-1-β-D-ribofuranosyl benzimidazole (DRB) sensitivity-inducing factor (DSIF) and a negative elongation factor (NELF). CDK9 is then recruited to promote transcriptional elongation via phosphorylation of the carboxy-terminal domains (CTDs) of RNA polymerase II, DSIF, and NELF to enable transcription elongation^7^. CDK9 is also a critical transcriptional regulator required for the efficient expression of most genes. Recent studies showed that inhibition of CDK9 is a potential therapeutic target for the anticancer activity. Flavopiridol and roscovitine have broad activities against CDK2, CDK7, and CDK9 that have been assessed in clinical anticancer clinical trials. They induce cell-cycle arrest and apoptosis in hematopoietic malignancies, including chronic lymphocytic leukemia (CLL) and multiple myelomas (MMs)^8–11^. Induction of apoptosis is associated with inhibition of transcription, and CDK9 is a critical mediator of this effect. The transcription inhibitor, DRB, principally inhibits CDK9 and also triggers apoptosis in leukemic cells and colon carcinoma cells^12,13^. Another CDK inhibitor, dinaciclib, was demonstrated to exert antitumor effects in MLL-rearranged acute myeloid leukemia through suppression of CDK9 activity^14^. Despite clinical development of multiple inhibitors with CDK9 activity, the precise mode of action driving antitumor effects has yet to be fully elucidated, although their preclinical activities have been attributed predominantly to causes down-regulation of the antiapoptotic protein myeloid cell leukemia 1 (Mcl-1) that is required for apoptosis induction^15,16^. Mcl-1 is an antiapoptotic, B-cell lymphoma (BCL)-2 family protein whose high expression has been associated with increased cancer cell survival that translates to chemotherapy resistance and poor patient prognoses^17,18^.

Apoptosis, a process of programmed cell death, is important for development and tissue homeostasis in multicellular organisms. Dysregulation of apoptosis is associated with a variety of human diseases, including developmental and immunological disorders, neurodegeneration, and cancers^19^. Apoptosis depends on activation of caspases that are synthesized as inactive pro-enzymes. Activated caspases initiate a protease cascade that cleaves vital cellular proteins leading to cell death^20^. Apoptosis can be initiated through two main pathways, extrinsic and intrinsic pathways. The extrinsic pathway acts through cell-surface death receptors, while the intrinsic pathway is mediated by intracellular Bcl-2 proteins^21^. The BCL-2 family consist of three groups of proteins based on Bcl-2 homology (BH) domains and function. They include the multi-domain antiapoptotic (e.g., Bcl-2, Bcl-xL, and Mcl-1), multi-domain proapoptotic (e.g., Bax and Bak), and BH3-only proapoptotic proteins (e.g., Bim, Bid, Bad, Puma, Noxa). Activation of Bax and Bak results in mitochondrial outer membrane permeabilization (MOMP), cytochrome C release and then apoptosis. Antiapoptotic proteins may prevent apoptosis by binding and inhibiting Bax and Bak. In response to intracellular apoptotic stimuli, only BH-3 proteins inhibit antiapoptotic proteins and activate multi-domain proapoptotic proteins. Alterations of Bcl-2 family proteins are common features in cancer cells and play a role in therapy resistance.

Breast cancer is the most common cancer and a major cause of cancer deaths in women in the Western world^22^. In Taiwan, the incidence and mortality of breast cancer have significantly increased in the past 2 decades^23^. Due to the important roles of CDK9 in malignancies, we found that the CDK9 inhibitor, DRB, also induced apoptotic death through rapid depletion of Mcl-1 in MCF-7 breast cancer cells and exerted a protective effect. Through searching potential signaling pathway that also target Mcl-1 expression, we identified that Akt-PI3K signaling and proteasome degradation are involved in the molecular mechanism of DRB that mediates apoptosis. Thus, a CDK9 inhibitor in combination with the AKT inhibitor, LY294002, or the proteasome inhibitor, MG-132, may provide a potentially therapeutic strategy to enhance the sensitivity of breast cancer cells to CDK9 inhibition.

## Materials and method

### Cell culture, transfection and treatment

The MCF-7 and T-47D human breast tumor cell line were gifts from Yuan-Ching Chang (Department of Surgery, Mackay Memorial Hospital, Taipei, Taiwan) and Liang-Chuan Lai (Graduate Institute of Physiology, National Taiwan University, Taipei, Taiwan) separately. The MCF-7 was maintained in Dulbecco’s modified Eagle medium (DMEM) supplemented with 10% fetal bovine serum (FBS) and 1 mM sodium pyruvate. T-47D was maintained in RPMI-1640 medium supplemented with 10% FBS. HEK-293 T cells were kindly provided by Li-Chung Hsu (Institute of Molecular Medicine, National Taiwan University, Taipei, Taiwan) and maintained in DMEM supplemented with 10% FBS. Cell transfections were performed by Turbofect (Thermo, Waltham, MA, USA) according to the manufacturer’s instructions. The Flag-Mcl-1 plasmid (Clone ID: OHu21277) was purchased from Genscript (Piscataway, NJ, USA). 5,6-dichloro-1-β-d-ribofuranosylbenzimidazole (DRB) and LY294002 were purchased from Sigma-Aldrich (St. Louis, MO, USA). MG-132 was purchased from Merck Millipore (Billerica, MA, USA). S63845 was purchased from Selleck Chemicals (Houston, TX, USA).

### MTT assay

Cells were seeded in 48-well plates at a density of 5 × 10^4^ cells/well. After overnight culture, cells were fed with 250 μl of fresh medium containing different concentrations of DRB. At different periods of time, 10 μl of 5 mg/ml MTT [3-(4,5-dimethylthiazol-2-yl)-2,5 diphenyl tetrasodium bromide] (Sigma-Aldrich) was added to each well. After 3 h of incubation at 37 °C, the medium was removed and cells were lysed with 62.5 μl of isopropanol containing 40 mM HCl, followed by incubation at 37 °C for 30 min to dissolve the crystals. The absorbance at 570 nm was measured using an enzyme-linked immunosorbent assay (ELISA) plate reader.

### Western blot analysis

Western blotting was performed as previously described^24^. Briefly, cells were lysed in modified RIPA buffer (50 mM Tris–HCl at pH 8, 150 mM NaCl, 5 mM EDTA, 1 mM EGTA, 1% NP-40, 0.5% sodium deoxycholate, 0.1% SDS, 5 mM DTT, 2 mM phenylmethylsulfonyl fluoride, and 10 μg/ml leupeptin) and incubated on ice for 30 min. After centrifugation (13,000 g for 10 min at 4 °C), the supernatant fraction was collected and the protein concentration of each sample was determined by the Bradford method (BioRad, Hercules, CA, USA). Equal amounts of protein were resolved by 8–12% SDS-PAGE, and then transfer to PVDF membranes. Blots were blocked with Blocking One (Nacalai, Kyoto, Japan) for 1 h and cut the membranes to incubate with a primary antibody in TBST (125 mM NaCl, 25 mM Tris–HCl at pH7.5, and 0.1% Tween 20) containing 5% Blocking One at 4 °C for overnight. After washing with TBST, blots were incubated with a horseradish peroxidase (HRP)-conjugated secondary antibody for 1 h at room temperature. Signals were detected by a chemiluminescence assay. The following antibodies were used in this study: Mcl-1, Bcl-xL, Akt, p53, and α-tubulin which were purchased from Santa Cruz Biotechnology (Dallas, TX, USA). Bax, Bcl-2, caspase-9, cleaved caspase-7, cleaved PARP, and phosphorylated (p)-AKT were purchased Cell Signaling (Boston, MA, USA). FLAG and β-actin were purchased from Sigma-Aldrich. RNA polymerase II, phospho-CTD Ser-2 (p-Ser-2), phospho-CTD Ser-5 (p-Ser-5), and GAPDH were purchased from Merck Millipore. PARP was obtained from BD Biosciences (San Jose, CA, USA). CDK9 were purchased from Abcam (Cambridge, UK).

### shRNA knockdown

Short hairpin (sh)RNA-expressing lentiviral plasmids (pLKO.1-shRNA) were purchased from the National RNAi Core Facility (Academia Sinica, Taipei, Taiwan). Mcl-1 was targeted with the TRCN0000005518 or TRCN0000005516 construct. shRNA targeting LacZ (TRCN0000072223) was used as a control. Lentiviral particles were prepared as described previously^25^. Briefly, the pLKO.1-shRNA construct was co-transfected with the package plasmids pCMV-ΔR8.91 and pMD.G into HEK293T cells using the TurboFect reagents (Fermentas, Waltham, MA, USA). After 24 h and 48 h of transfection, the supernatant was collected and 0.4 μm filtered. Targeted cells were incubated with the viral supernatant in the presence of 8 μg/mL polybrene (Sigma-Aldrich). After 48 h of transduction, cells were maintained in selection medium containing puromycin (5 μg/mL) for 3 days before use.

### Flow cytometry analysis

A cell apoptosis analysis was performed using an FITC Annexin V Apoptosis Detection Kit (BioVision, Waltham, MA, USA) according to the manufacture’s instruction. Cells were harvested, washed with cold PBS and resuspended in binding buffer. Cells were stained with Annexin V-FITC and propidium iodide (PI) for 15 min at 25 °C in the dark. Stained cells were analyzed with flow cytometry within 1 h. Data were recorded by BD LSRFortessa (BD Biosciences).

### DNA fragmentation analysis

MCF-7 cells (4 × 10^5^) seeded in six-well plates were pretreated with or without MG-132 and then exposed to DRB as indicated. Cells were harvested and resuspended in 40 μl of lysis buffer (10 mM Tri-HCl at pH 7.5, 1 mM EDTA and 0.25% Triton X-100), followed by adding 10 μl RNase A (10 mg/ml) and incubated at 37 °C for 20 min as previously described^26^. Then, 5 μl of proteinase K (20 mg/ml) was added to the cell lysate and incubated at 37 °C until the solution cleared. DNA was isolated using a QIAquick PCR Purification Kit (Qiagen, Germantown, MD, USA) and analyzed by 2% agarose gel electrophoresis.

### Statistical analysis

Statistical analyses were carried out by the Student’s *t*-test using GraphPad Prism 6 software (GraphPad Software, San Diego, CA, USA). Values of *p* < 0.05 were considered statistically significant.

## Results

### CDK9 inhibition leads to enhanced apoptosis of breast cancer cells

**Figure 1:**
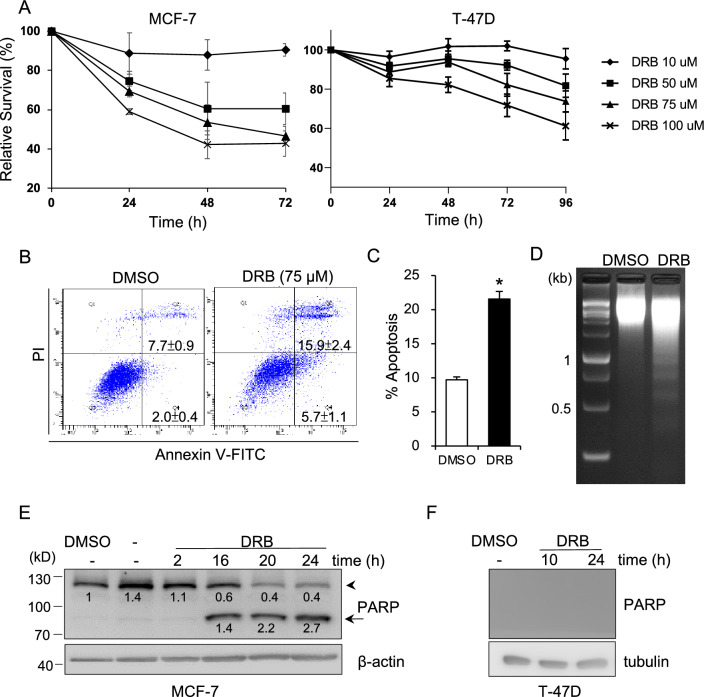
DRB induces apoptotic death in MCF-7 and T-47D cells. (**A**) MCF-7 and T-47D cells were treated with different concentrations of DRB. At the indicated times, cell viability was assayed by an MTT analysis. (**B**) MCF-7 Cells were treated with DRB (75 μM) or control DMSO for 24 h. Cells were labeled with Annexin V-FITC/PI and analyzed by flow cytometry. Annexin V + /PI- cells were the early apoptotic fraction in the lower right, and Annexin V + /PI + cells were the late apoptotic fraction in the upper right. A representative result from three experiments is shown. (**C**) The ratio of apoptosis was the percentage of early apoptosis plus late apoptosis. Data are the means ± SEM. (**D**) MCF-7 Cells were treated with 75 μM DRB or control DMSO for 16 h. DNA was isolated for gel electrophoresis. (**E**) MCF-7 or (**F**) T-47D Cells were treated with DRB (75 μM) or control DMSO for the indicated times. Cells were lysed and subjected to Western blotting for PARP. The arrowhead indicates PARP and the arrow indicates cleaved PARP. The band densities of the PARP and cleaved PARP were quantitated, normalized to β-actin and PARP, respectively, and the mean values associated with each band are shown below.

To investigate the in vitro anti-cancer efficacy of the CDK9-specific inhibitor, DRB, MCF-7 and T-47D breast cancer cells were exposed to various concentrations of DRB for up to 72 h, and cell viability was determined by MTT assays. As shown in Fig. 1A, dose-dependent cell-growth inhibition was observed in DRB-treated MCF-7 and T-47D cells. MCF-7 cell viability decreased to 50% at after 72 h at a concentration of 75 μM, whereas no significant increment in cell viability was detected after 100 μM DRB treatment compared to the control (Fig. 1A). Similar result was found in T-47D cells (Fig. 1A). These results showed that DRB inhibited the cell growth of breast cancer cells in dose- and time-dependent manners. To further examine whether cells underwent apoptosis, DMSO- (control) or DRB-treated MCF-7 cells were stained with Annexin V and PI. A flow cytometric analysis of stained cells can distinguish cells into two groups, namely early apoptosis (Annexin V + , PI-) and late apoptosis (Annexin V+ , PI +). As shown in Fig. 1B, DRB exposure at a concentration of 75 μM resulted in a higher early apoptotic population (5.7 ± 1.1 vs. 2 ± 0.4%) and late apoptotic population (15.9 ± 2.4 vs. 7.7 ± 0.9%) compared to the control. Our flow cytometric analysis showed that the proportion of apoptotic cells significantly increased two fold following DRB treatment of MCF-7 cells (Fig. 1C). Correspondingly, DRB-induced apoptotic cell death was also confirmed by the appearance of DNA fragmentation (Fig. 1D) and cleavage of PARP at 16 h (Fig. 1E). However, cleavage of PARP was not found in T-47D cells followed by DRB treatment (Fig. 1F). Together, our results demonstrated that DRB resulted in MCF-7 breast cancer cell death through an apoptotic pathway.

### DRB downregulates Mcl-1 expression in breast cancer cells

**Figure 2:**
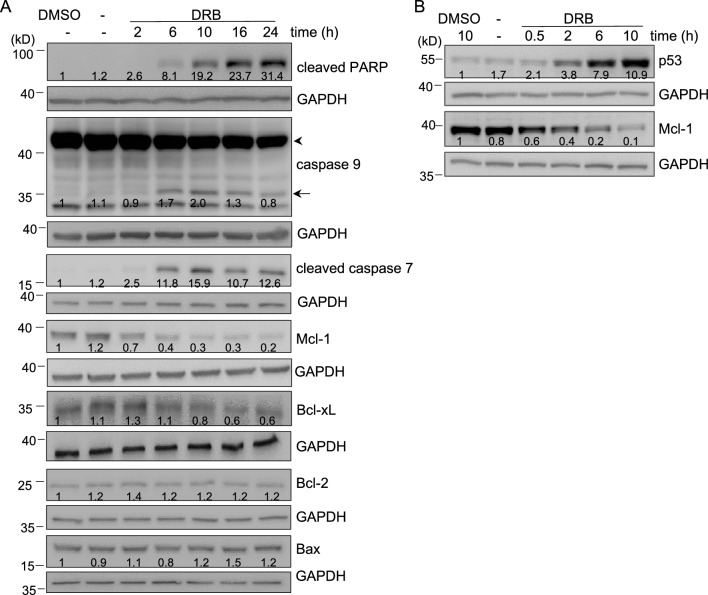
Expression of BCL-2 family proteins in DRB-treated MCF-7 cells. MCF-7 cells were treated with DRB (75 μM) or control DMSO for the indicated times. Cells were lysed and subjected to Western blotting with the indicated antibodies. The arrowhead indicates procaspase-9 and the arrow indicates cleaved caspase-9. The band densities of the western blots were quantitated, normalized to individual GAPDH, and the mean values associated with each band are shown below.

Since activation of caspase-dependent pathways plays a key role in the execution of apoptosis, we next examined expression levels of cleaved caspases and the downstream PARP molecule after DRB treatment by a Western blot analysis. As shown in Fig. 2A, the cleavage of caspase-9 and caspase-7 significantly increased from 6 to 10 h, and remained high even after 24 h of treatment. Consistently, PARP cleavage in MCF-7 cells was also detected 6 h after DRB treatment and continued to increase to 24 h. Our data suggested that an intrinsic caspase pathway and PARP inactivation were involved in DRB-mediated apoptosis (Fig. 2A).

We further examined if the DRB initiated apoptosis by affecting antiapoptotic molecules, and a Western blot analysis showed that 0.5 h of DRB treatment caused a significantly reduction in Mcl-1 protein levels in a time-dependent manner (Fig. 2A and B). In contrast, other antiapoptotic proteins such as Bcl-2, Bcl-xL, and Bax, remained unaffected through 24 h of treatment (Fig. 2A). As also shown in Fig. 2B, the p53 protein accumulation markedly increased after 6 h of DRB treatment, indicating that the induction of p53 contributed to DRB-induced apoptosis in MCF-7 cells. Overall, these results indicated that the intrinsic apoptosis pathway elicited by DRB in breast cancer cells results was mediated by Mcl-1 and p53.

### DRB inhibits CDK9 activity

**Figure 3:**
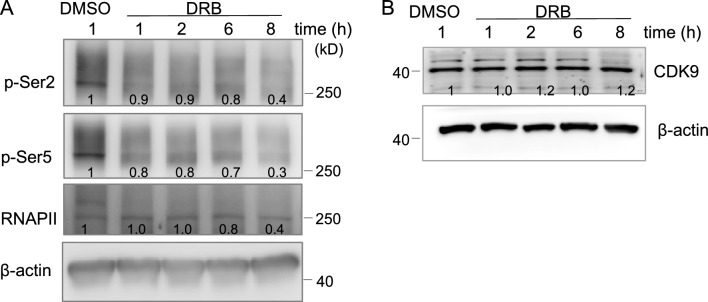
Effects of DRB on the phosphorylation of the RNA polymerase II CTD in MCF-7 cells. MCF-7 cells were treated with DRB (75 μM) or control DMSO for the indicated times. Cells were lysed and subjected to Western blotting for RNA polymerase II CTD, phosphorylated CTD Ser2/Ser5 and CDK9. The band densities of the western blots were, quantitated, normalized to individual β-actin, and the mean values associated with each band, are shown below.

As DRB is a selective inhibitor of the CDK9 kinase, we thus asked whether DRB-mediated apoptosis involves inactivation of CDK9. We investigated the effects of DRB on the phosphorylation of the CTD of RNAPII at both Ser^2^ and Ser^5^ sites, which was proposed to block transcriptional elongation and consequently eliminate gene production. A Western blot analysis of MCF-7 cells treated with 75 μM of DRB at different time points showed that both phosphorylation of both Ser^2^ and Ser^5^ was diminished in a time-dependent manner after 1 h of DRB treatment (Fig. 3A), but no effect on CDK9 expression level (Fig. 3B). Thus, the above data implied that DRB has a negative role in CDK9-mediated phosphorylation of RNAPII.

### Inhibition of Mcl-1 is sufficient to mimic DRB-induced apoptosis

**Figure 4:**
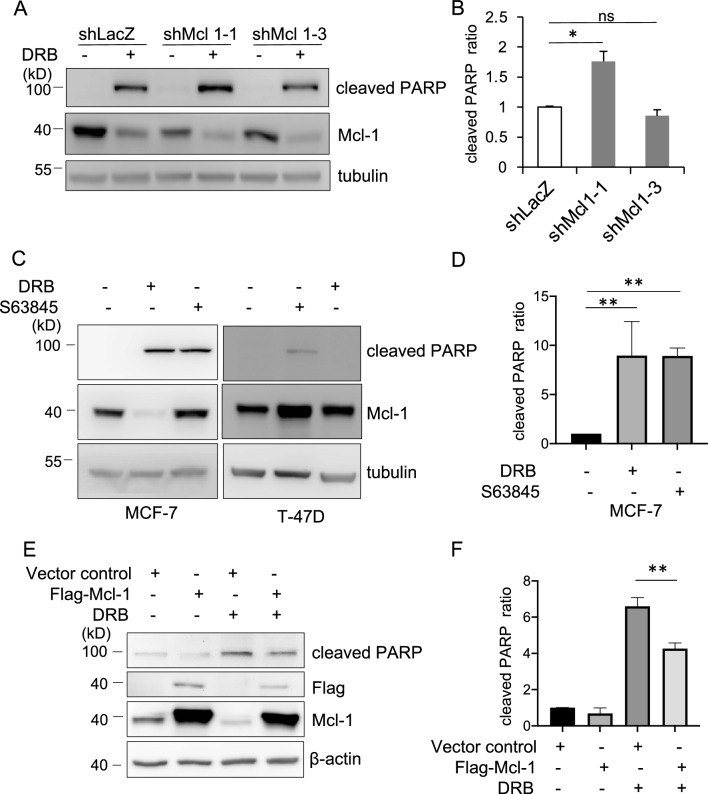
Loss of Mcl-1 contributes to DRB- mediated apoptosis in MCF-7 cells. (**A**) Cells were transduced with shRNA specific for Mcl-1 or the LacZ control and then treated with DRB for 16 h. Cells lysates were subjected to Western blotting with the indicated antibodies. The band intensity of the cleaved PARP was quantified by densitometry, normalized over the loading control and presented as fold induction over the control in (**B**). (**C**) MCF-7 and T-47D cells were treated with DRB or S63845. Cells lysates were then subjected to Western blotting for PARP and Mcl-1. The band intensity of the cleaved PARP was normalized to tubulin and presented as fold induction over the control in (**D**). (**E**) Cells were transfected with Flag-Mcl-1 or the vector control and then treated with DRB or DMSO for 10 h. Cell lysates were subjected to Western blotting for the indicated antibodies. The band densities of the western blots were quantitated, normalized to β-actin, and the mean values associated with cleaved PARP are shown in (**F**). Data are the means ± SEM. *p < 0.05, ** *p* < 0.01. Data represent a minimum of 3 independent experiments.

To investigate the role of Mcl-1 in regulating the life and death of cancer cells, we carried out shRNA knockdown, inhibition and overexpression experiments in MCF-7 cells. Specific knockdown of Mcl-1 expression by RNA interference (shMcl 1–1, but not shMcl 1–3) increased levels of PARP cleavage in response to DRB, indicating that loss of Mcl-1 resulted in induction of apoptosis in MCF-7 cells (Fig. 4A and B). Similar to shMcl-1, inhibition by selective Mcl-1 inhibitor, S63845, also significantly increased the cleavage PARP in both MCF-7 and T-47D cells (Fig. 4C and D). To further examine the role of Mcl-1 in DRB-mediated apoptosis, Mcl-1 was introduced into MCF-7 cells by transient transfection of plasmids expressing the Flag-tagged Mcl-1 protein. Flag-epitope expression of the Mcl-1 protein resulted in the effect of DRB on PARP cleavage being dramatically reduced, which caused significant resistance to DRB-induced apoptosis (Fig. 4E and F). This line of evidence indicated that downregulation of Mcl-1 is an important mechanism of DRB-induced apoptosis in MCF-7 cells.

### The PI3K-AKT pathway is involved in DRB-induced apoptosis

**Figure 5:**
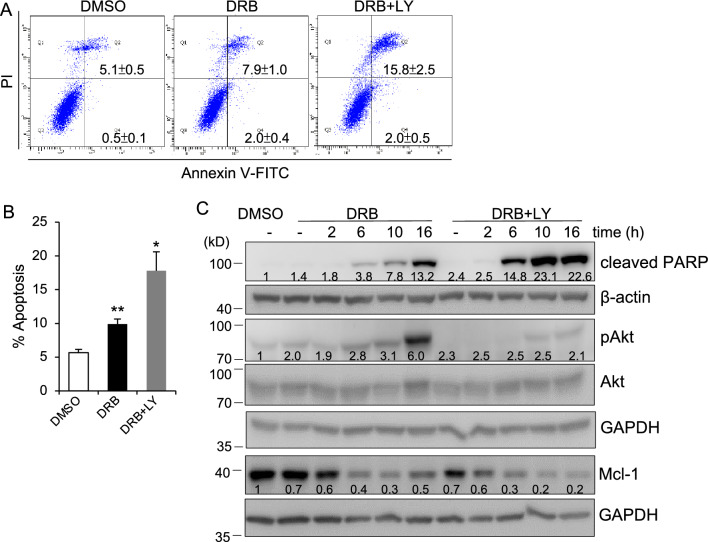
PI3K inhibition enhances DRB-induced apoptosis in MCF-7 cells. (**A**) MCF-7 cells were pretreated with the PI3K inhibitor LY294002 (20 μM), or control DMSO for 30 min and then exposed to DRB (75 μM) or control DMSO for 24 h. Cells were labeled with Annexin V-FICT/PI and analyzed by flow cytometry. A representative result from three experiments is shown. (**B**) Data are the means ± SEM of three independent experiments. (**C**) Cells were pretreated with or without the PI3K inhibitor, LY294002 (20 μM), for 30 min and then exposed to DRB (75 μM) or control DMSO for the indicated times. Cells were lysed and subjected to Western blotting for the indicated antibodies. β-actin and GAPDH was used as loading controls. The band densities of the western blots were quantitated and the mean values associated with each band are shown below.

Activation of PI3K-AKT signaling is the most important intracellular pathways, which contributes to the tumor development and resistance to anticancer therapies. To establish the role of PI3K-AKT in DRB-induced apoptosis, MCF-7 cells were pretreated with the PI3K inhibitor LY294002 (LY), for 30 min before DRB exposure and then incubated for 24 h. The flow cytometric analysis showed that MCF-7 cells underwent significant apoptosis upon LY pretreatment combined with DRB, compared to DRB treatment alone (Fig. 5A and B). We next examined the effects of DRB on the activation of AKT, a major downstream effector of PI3K, by a Western blot analysis. Results showed that phosphorylation of AKT (Ser473) was significantly elevated at 16 h in DRB-treated cells compared to control cells, which correlated with an increase in the cleavage of PARP increase and a decrease in Mc1-1 (Fig. 5C). Furthermore, LY pretreatment attenuated phosphorylation of AKT, accompanied by significantly augmentation of the levels of PARP cleavage and diminished Mcl-1 expression relative to cells only treated with DRB (Fig. 5C). Taken together, these results indicated that AKT-PI3K acted as a negative regulator involved in DRB-induced apoptosis.

### Inhibition of the proteasome enhances DRB-induced apoptosis

**Figure 6:**
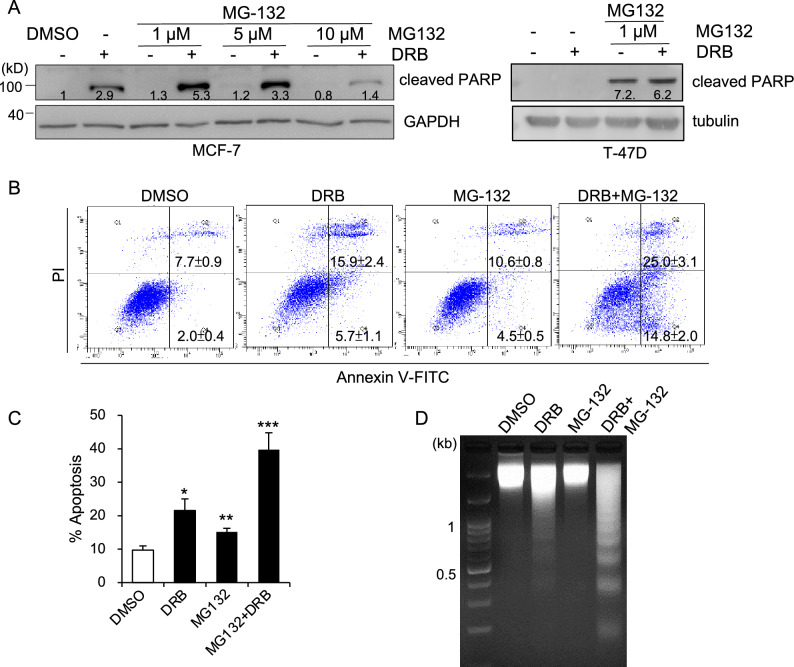
DRB combined with the proteasome inhibitor MG-132 triggers enhanced apoptosis. (**A**) MCF-7 or T-47D cells were pretreated with or without the proteasome inhibitor, MG-132, at the indicated concentrations for 30 min and then exposed to DRB (75 μM) or control DMSO for 16 h. Cells were lysed and subjected to Western blotting for cleaved PARP. The band densities of the cleaved PARP were quantitated and the mean values associated with each band are shown below. (**B**) Cells were treated with DRB (75 μM), MG-132 (1 μM), a combination of DRB and MG-132, or control DMSO for 24 h. Cells were labeled with Annexin V-FICT/PI and analyzed by flow cytometry. A representative result from three experiments is shown. (**C**) The ratio of apoptosis is shown as the means ± SEM. (**D**) Cells were treated as in B for 16 h followed by DNA isolation for gel electrophoresis.

PARP is known to be a target of proteasomal degradation^27^. To further investigate the influence of proteasome inhibition in DRB-induced apoptosis, MCF-7 cells were pretreated with varying concentrations of the proteasome inhibitor, MG-132, for 30 min before exposure to DRB. The combination of MG-132 and DRB induced massive cleavage of PARP at low concentrations of 1 μM and 5 μM, but not at 10 μM (Fig. 6A). In T-47D cells, the combination of 1 μM MG-132 and DRB induced cleavage of PARP was similar to MG-132 alone (Fig. 6A). Also, the flow cytometric analysis showed that the co-treatments of MCF-7 cells with DRB and 1 μM MG-132 caused a significant increase in the apoptotic cell population compared to DRB only treatment (Fig. 6B and C). Consistently, DNA fragmentation prominently increased with combined MG-132 and DRB treatment compared to DRB or MG-132 alone (Fig. 6D). These results indicated that the effect of MG-132 on DRB-induced apoptosis was mediated by prevention of cleaved PARP degradation.

## Discussion

Breast cancer is a very common malignancy and a leading cause among women in most developed countries^28^. It is a heterogeneous disease, which comprises many subtypes with different biological features that lead to differences in responses to various treatment and clinical outcomes^29,30^. Researchers have divided breast cancer into four main molecular subtypes: luminal A (estrogen receptor (ER)+ , progesterone receptor (PR)+ , human epidermal growth factor receptor 2 (HER2)-), luminal B (ER+ , PR−), HER2 positive, and triple negative. About 70% of all breast cancers are “ER-positive”, which means that cancer cells grow in response to the hormone estrogen. ER-positive breast cancers have the most favorable prognosis among the four groups and typically respond to endocrine therapies, such as tamoxifen and aromatase inhibitors, or targeted endocrine-related therapies, such as PI3K-AKT-mTOR signaling, CDKs, epigenetic regulators and selective estrogen down-regulator (SERD)-selective estrogen receptor covalent antagonists^31^. Unfortunately, however, metastatic recurrences and resistance to endocrine therapy commonly occurred in 30% of patients who receive primary therapy, highlighting the pressing need for more-effective therapies for this class of ER-positive breast cancers^32,33^.

Apoptosis, as a form of programmed cell death, is regulated by many factors that affect functions of intracellular genes and related proteins. Most stimulations induce the apoptotic process via the mitochondrial pathway, because it consists of the main signal in the apoptotic process^34–36^. In general, the membrane potential of mitochondria is greatly reduced and cytochrome C is released from the mitochondria to the cytoplasm and then activates caspase activity in the cytoplasm^37^. For example, cytochrome C can bind apoptotic protease-activating factor (Apaf)-1 that forms the apoptosome which activates caspase-9. Once active, caspase-9 can directly cleave and activate caspase-3 and caspase-7. Caspase-3, as the primary executioner of apoptotic death, initiates cell death and caspase-7 can cause an accumulation of reactive oxygen species (ROS) production and functions to detach cells from the extracellular matrix (ECM)^34^. Many regulatory molecules of the BCL-2 family are commonly involved in cell apoptosis, such as pro-apoptotic proteins (Bad, Bax, and Bid) and antiapoptotic proteins (Bcl-2, BCL-xl, and Mcl-1)^38^. The balance between these two groups of proteins regulates the progress of apoptosis and determines the fate of tumor cells. In this study, we used Annexin V-FITC and PI staining to detect cell apoptosis and observed that both MCF-7 and T-47D cell apoptosis rate increased with an increasing DRB concentration. We found that MCF-7 cells were more sensitive to DRB than T-47D cells since only 50% of cell viability detected 24 h after expose to 75 μM DRB, whereas there was 80% in T-47D cells. It is possible due to anti-apoptosis mechanisms, including activation of different caspases and mitochondrial changes, are more strongly expressed in T-47D than in MCF-7^39,40^. In addition, DRB induced apoptosis by downregulating Mcl-1, and activating apoptosis-related proteins, including cleaved caspase-7, caspase-9, and PARP. T-47D cells had higher survival rate at 48 h than 24 h probably due to significant plasma membrane changes occurred later. It is possible that binding of annexin-V in T-47D cells after DRB treatment and continued to 24 h, however this occurred later where significant annexin-V binding was achieved 48 h after 10–75 μM DRB^39^. These findings are consistent with reports that DRB induces apoptosis in other tumor cells, such as acute myeloid leukemia, human laryngeal carcinoma and colon carcinoma cells, which provided additional insights for exploring the antiproliferation mechanism of DRB in breast cancer^12,13,41^.

Frequent overexpression of Mcl-1 in various human tumors is recognized as a defense mechanism for cancer cells to evade apoptosis^42–45^. Nearly 36% of breast cancer cases among 3131 cancer specimens exhibited increased levels of Mcl-1 expression levels^46^. Also, high levels of Mcl-1 expression are often associated with poor prognosis and resistance to chemotherapeutic drugs. For example, breast cancer, especially ER-positive breast cancer, is characterized by amplification of Mcl-1 expression, which was correlated with a high tumor grade and poor prognoses in patients^47^. Also, breast cancer cells are resistant to clinical trial drugs such as the Bcl-2/Bcl-xL inhibitor, ABT-263, which may result from increased Mcl-1 expression^48^. Herein, we found that downregulation of Mcl-1 expression increased ER-positive breast cancer MCF-7 cell sensitivity to DRB treatment. In contrast, overexpression of Flag-epitope Mcl-1 partially reversed the effects of DRB on the apoptotic process. Since short half-life is one of the unique features of Mcl-1 in the Bcl-2 family, it is possible the reason why we didn’t observe fully rescue cells from apoptosis after Mcl-1 overexpression^49^. Recently, amplification of *Mcl-1* gene loci was also found in triple-negative breast cancer, and NOXA, an endogenous Mcl-1 inhibitor, depressed HER2-positive breast cancers, suggesting that DRB could be further explored as treatment strategies for ER-negative breast cancer^18,50^.

Mcl-1 exerts its antiapoptotic function by sequestering the proapoptotic proteins BAK/BAX to avoid the initiation of the apoptotic program, thereby allowing cells to maintain homeostasis. Upon apoptotic signals, activated BH3-only proteins can bind to Mcl-1 antiapoptotic proteins, which allows the release of BAK/BAX^51^. After dissociation from Mcl-1, BAK/BAX are translocated into the outer membranes of mitochondria which leads to the release of cytochrome C and the activation of initiator caspases^52,53^. In the present study, downregulation of the antiapoptotic proteins, Mcl-1 and Bcl-xL, was observed following DRB stimulation. Thus, it is possible that the loss of Mcl-1 and Bcl-xL could accelerate the release of BAK/BAX and cytochrome C to activate the apoptotic progress. However, the detailed mechanism needs to be further investigated in the future.

The PI3K-AKT pathway regulates many normal cellular processes including cell proliferation, survival, growth, and motility, which all are critical for tumorigenesis^54^. Aberrant activation of the PI3K-AKT pathway was found in nearly 30% of human cancers^55^. More than 20% of PI3KCA mutations were frequently identified in primary breast tumors and cell lines, including MCF-7 cells^56^. Since PI3KCA is an attractive marker, it has been considered a promising therapeutic target for breast cancer. So far, there are several PI3K inhibitors entering clinical trials, such as BEZ235 and GDC-0980, but drug toxicity is a major factor limiting their usage and treatment^57^. Thus, Vora et al. developed the combination therapy of GDC-0980 with CDK4/6 inhibitors to avoid drug toxicity and found that CDK4/6 inhibitors exacerbated GDC-0980-induced apoptosis of MCF-7 cells through inactivating the tumor suppressor proteins, retinoblastoma (Rb)^58^. Furthermore, flavopiridol, a CDK7/9 inhibitor, caused substantial phosphorylation of AKT in human glioblastoma cells, indicating that inhibition of CDKs might result in PI3K-AKT pathway activation, which may lead to cell survival or protection from apoptosis^59^. Similar to previous studies, we demonstrated that DRB induced AKT phosphorylation with consequent activation of the PI3K-AKT pathway, which may paradoxically confer resistance to the human MCF-7 breast cancer cell line. Treatment with the PI3K inhibitor, LY294002, could enhance DRB-induced apoptosis in MCF-7 cells. This finding may provide useful insights into the mechanism that confers resistance to human malignant cells. Since the cause of DRB-induced phosphorylation of AKT is still not known, we suggest that various types of human primary malignant cells should be examined to determine whether DRB treatment might induce the phosphorylation of AKT and development of resistance.

Previous studies reported that different concentrations of MG-132 had various effects on apoptosis depending on the sensitivity of the cellular response to MG-132. For example, the MG-132 IC_50_ for cell death in cervical cancer cells was estimated to be 5 μM, while the IC_50_ in lung cancer cells was 20 μM^60,61^. High doses of MG-132 induced apoptosis in leukemia cells via inhibition of NF-κB activity and increased ROS, but low doses caused no significant effect on cell death. Furthermore, a combination of low-dose (1 μM) MG-132 and doxorubicin enhanced the apoptosis in doxorubicin-resistant leukemia cells^62^. In addition, studies also found that a low dose (300 nM) of MG-132 combined with flavopiridol could induce apoptosis of leukemia cells by reducing the amounts of XIAP and Mcl-1 and the activity of NF-κB^63^. In the present study, we found that 1 μM MG-132 enhanced the cleavage of PARP and DRB-induced apoptosis of MCF-7 cells, suggesting that the mechanism may be related to reduction of NF-κB activity and antiapoptotic proteins.

A number of CDK9 inhibitors have been utilized as the therapeutic potential for anti-cancer drugs, and some of them have entered different phases of clinical trials^64,65^. Here, our data highlight the potential for development and use of selective inhibitor of CDK9, DRB, in treating ER-positive breast cancer through inhibition of Mcl-1. One important reason for the development of DRB and other highly specific CDK9 inhibitors, like AZD4573 and LDC000067, is that pan-CDK inhibitors such as flavopiridol exhibit a significant and dose-limiting level of cytotoxicity, possibly due to inhibition of multiple CDK families^66,67^. More broadly our data also suggest that targeting PI3K-AKT pathway or proteasomal degradation in combination with DRB might be an alternative approach in Mcl-1 upregulation cancer types, including acute myeloid leukemia, hepatocellular carcinoma, non-small cell lung cancer, breast cancer, and other Mcl-1 associated with resistance of tumor cells^49^. This will need to be pursued through preclinical models including cell line-based or patient-derived xenograft studies in mice.

In summary, DRB induced the apoptosis of human MCF-7 breast cancer cells by regulating Mcl-1 and Bcl-xL, and activating caspase family members in a time- and dose-dependent manners. Combining DRB with drugs targeting several escape pathways, such as PI3K-AKT and the proteasome degradation pathway, could be alternative strategies to prevent and block all the tumor survival escape routes. These results not only provide a potential drug for breast cancer treatment but also serve as a solid theoretical basis for understanding the development of the disease.

### Supplementary Information

Supplementary Information.

## Data Availability

All data generated or analysed during this study are included in this published article and its supplementary information files.
