# Supplementary material for: 5,6-Dichloro-1-β-D-ribofuranosylbenzimidazole (DRB) induces apoptosis in breast cancer cells through inhibiting of Mcl-1 expression
Source: Sci Rep. 2023 Aug 3;13:12621. doi: 10.1038/s41598-023-39340-x (PMC10400577; doi:10.1038/s41598-023-39340-x)
Supplement: Supplementary file 1 — Supplementary Information. [file 41598_2023_39340_MOESM1_ESM.pdf]

E

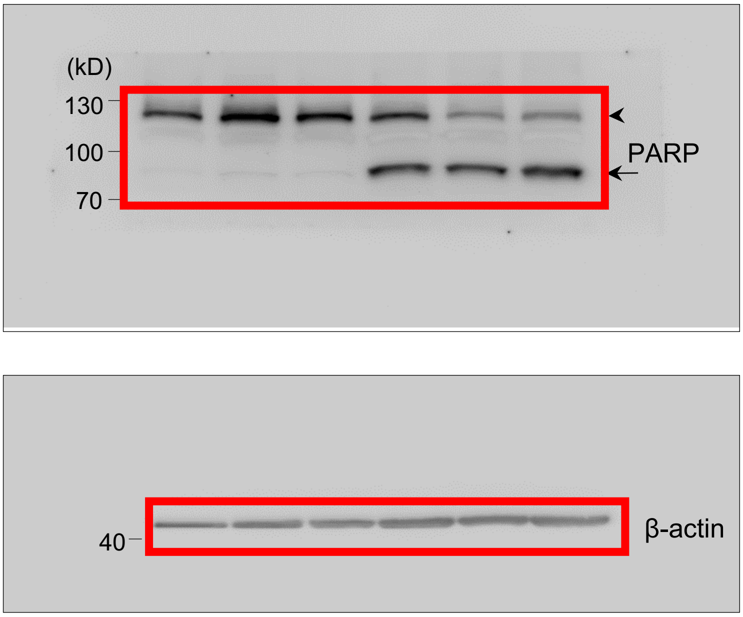

Supplementary Figure S1. Original images of blots presented in the main Figure 1.

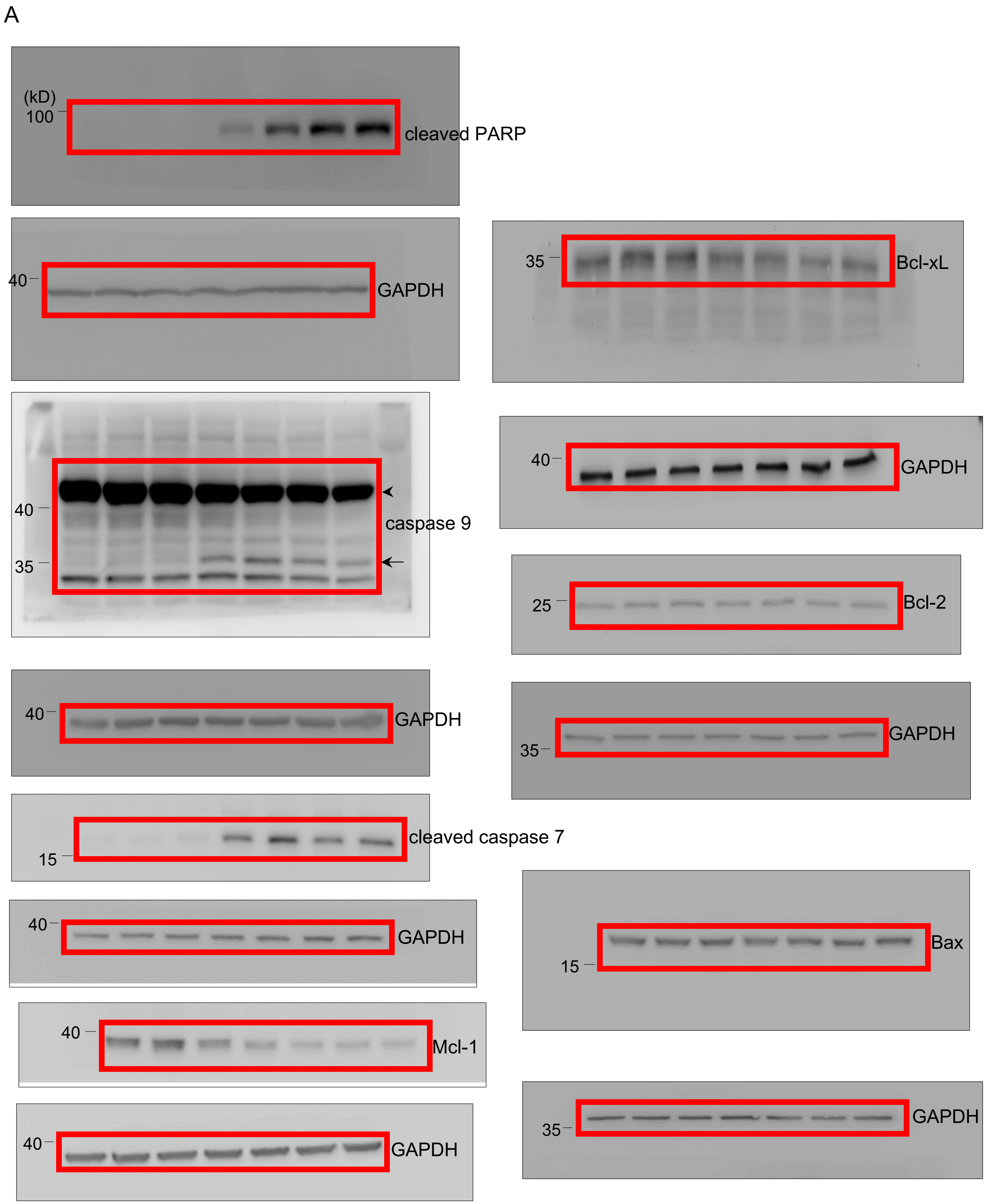

Supplementary Figure S2. Original images of blots presented in the main Figure 2A.

B

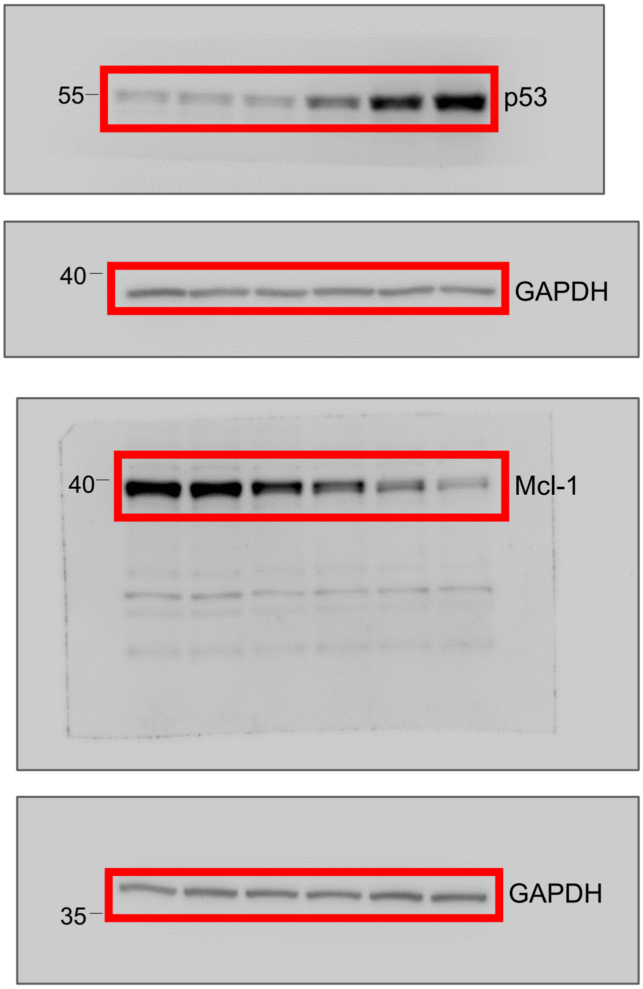

Supplementary Figure S3. Original images of blots presented in the main Figure 2B.

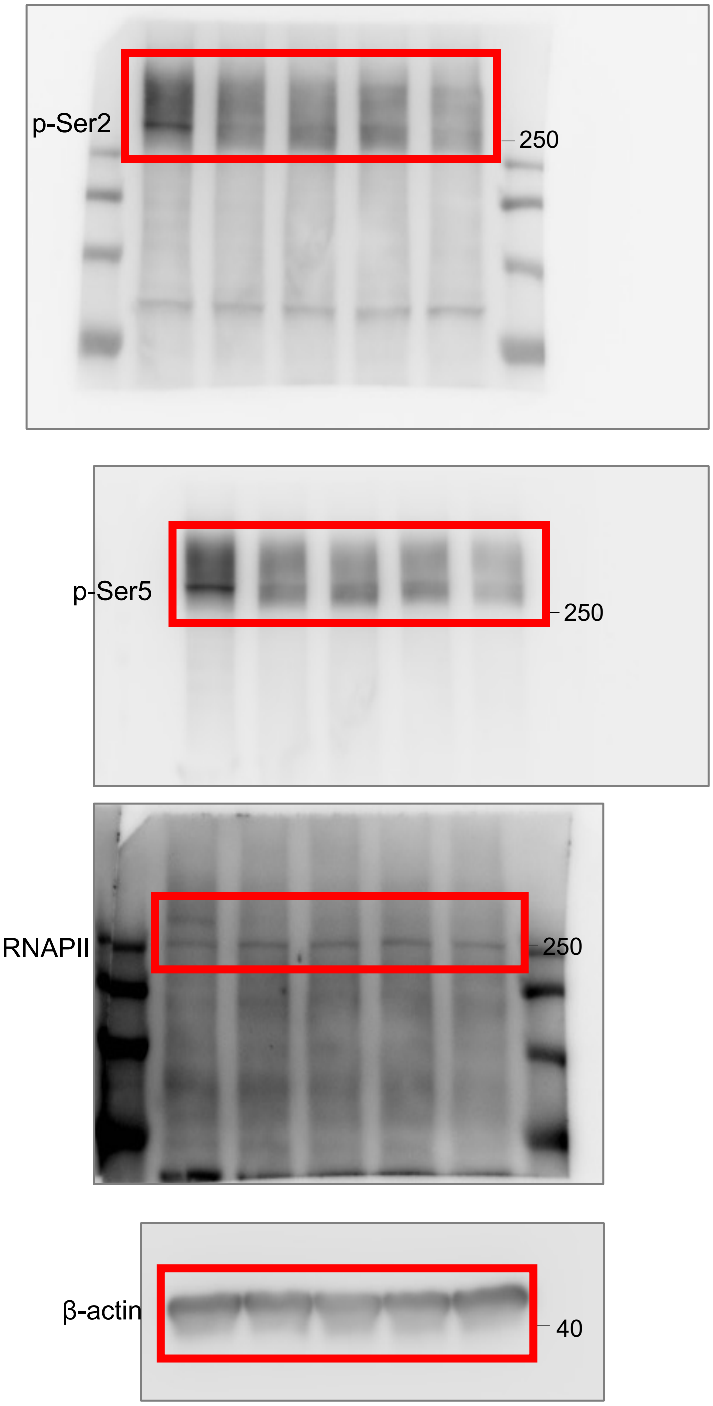

Supplementary Figure S4. Original images of blots presented in the main Figure 3A.

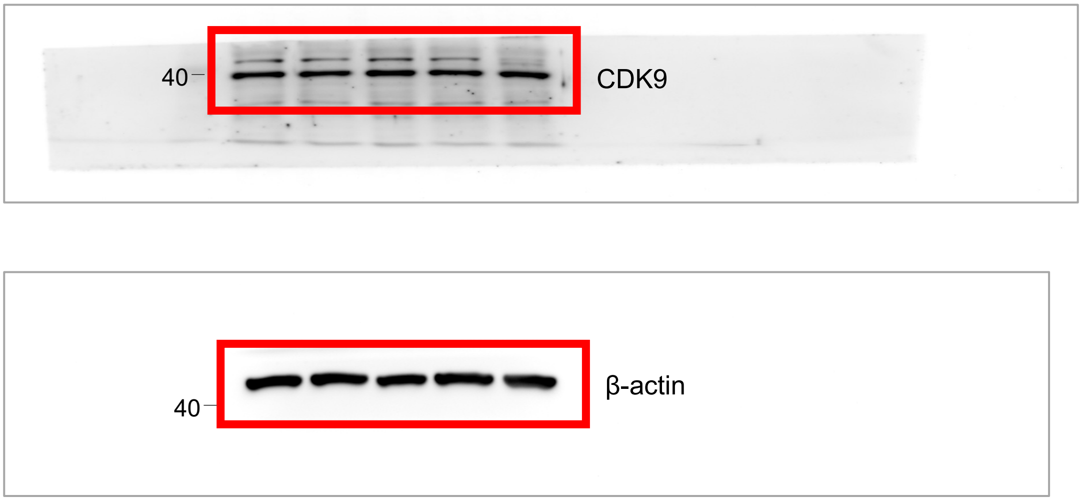

Supplementary Figure S5. Original images of blots presented in the main Figure 3B.

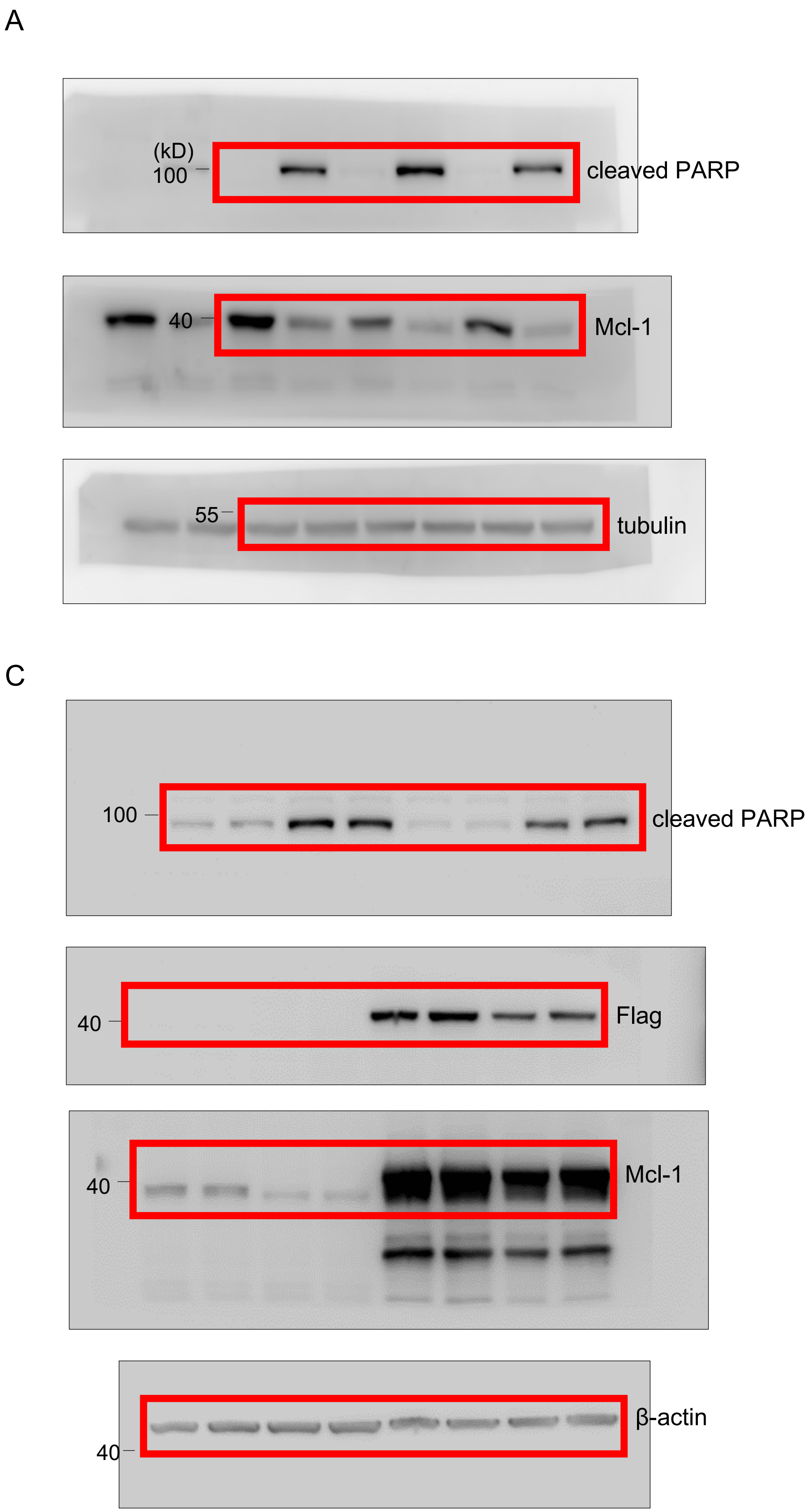

Supplementary Figure S6. Original images of blots presented in the main Figure 4.

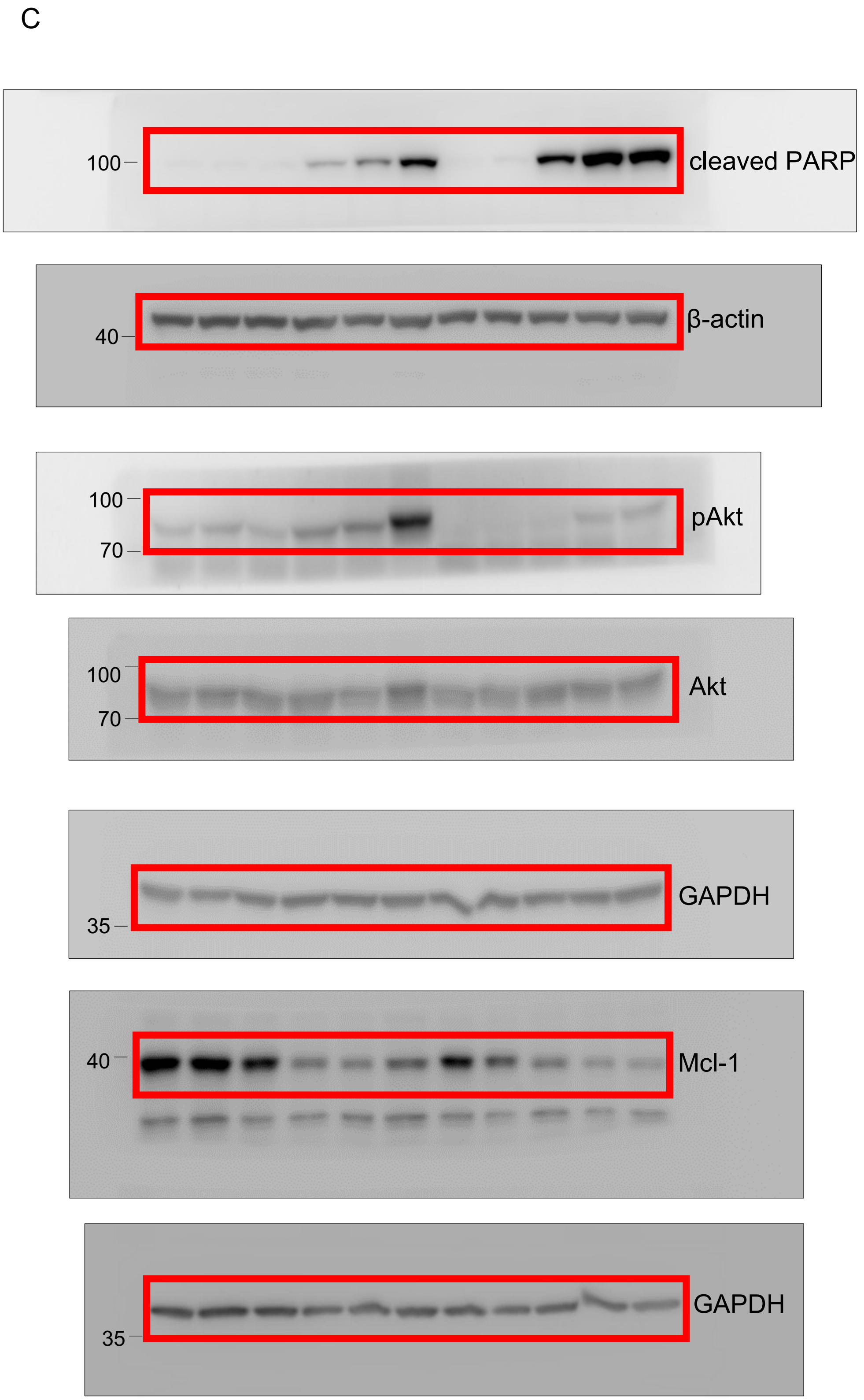

Supplementary Figure S7. Original images of blots presented in the main Figure 5.

A

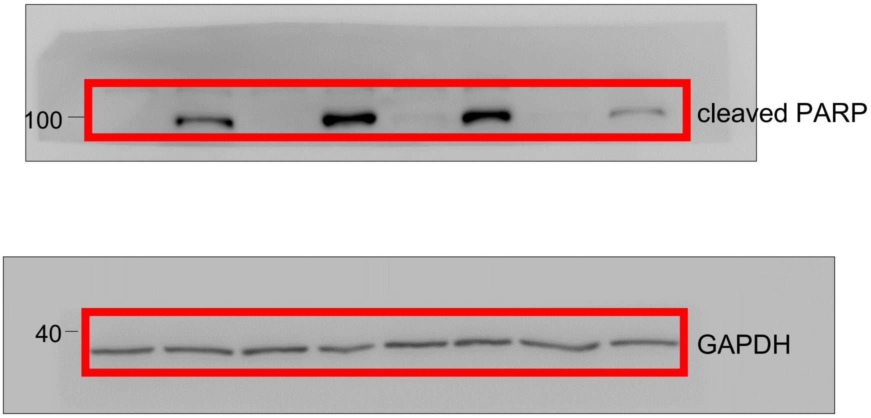

Supplementary Figure S8. Original images of blots presented in the main Figure 6.
